# Review: Subjective Time Perception, Dopamine Signaling, and Parkinsonian Slowness

**DOI:** 10.3389/fneur.2022.927160

**Published:** 2022-07-08

**Authors:** Edison K. Miyawaki

**Affiliations:** ^1^Department of Neurology, Mass General Brigham, Boston, MA, United States; ^2^Harvard Medical School, Boston, MA, United States

**Keywords:** parkinsonism, dopamine, subjective perception, time estimation, reward prediction

## Abstract

The association between idiopathic Parkinson's disease, a paradigmatic dopamine-deficiency syndrome, and problems in the estimation of time has been studied experimentally for decades. I review that literature, which raises a question about whether and if dopamine deficiency relates not only to the motor slowness that is an objective and cardinal parkinsonian sign, but also to a compromised neural substrate for time perception. Why does a clinically (motorically) significant deficiency in dopamine play a role in the subjective perception of time's passage? After a discussion of a classical conception of basal ganglionic control of movement under the influence of dopamine, I describe recent work in healthy mice using optogenetics; the methodology visualizes dopaminergic neuronal firing in very short time intervals, then allows for correlation with motor behaviors in trained tasks. Moment-to-moment neuronal activity is both highly dynamic and variable, as assessed by photometry of genetically defined dopaminergic neurons. I use those animal data as context to review a large experimental experience in humans, spanning decades, that has examined subjective time perception mainly in Parkinson's disease, but also in other movement disorders. Although the human data are mixed in their findings, I argue that loss of dynamic variability in dopaminergic neuronal activity over very short intervals may be a fundamental *sensory* aspect in the pathophysiology of parkinsonism. An important implication is that therapeutic response in Parkinson's disease needs to be understood in terms of short-term alterations in dynamic neuronal firing, as has already been examined in novel ways—for example, in the study of real-time changes in neuronal network oscillations across very short time intervals. A finer analysis of a treatment's network effects might aid in any effort to augment clinical response to either medications or functional neurosurgical interventions in Parkinson's disease.

## Introduction

Early in a parkinsonian syndrome, a family member or someone in regular contact with the patient often reports unmistakable slowing in movement, typically over a span of months or years. The patient herself may not be aware of any significant change in speed affecting activities of life, such that the diagnosis of parkinsonism may come as a surprise, met with skepticism of the diagnosis. The quantification of motor slowness in parkinsonism has been the subject of many types of investigation (1). But such objective measurement either may not or may only indirectly address the subjective experience of time's passing—what has been termed an individual's temporal cognition (2) or time perception (3). This paper examines subjective time perception in parkinsonism in light of observations of neuronal activity in substantia nigra pars compacta (SNc) during tasks, as assessed by fiber photometry of genetically defined dopaminergic neurons (DANs). The animal data introduce a consideration that has been variously addressed in human studies: do patients with idiopathic Parkinson's disease (PD) have a disease-related perturbation of their own moment-to-moment perception of time?

## Dopamine (DA), Basal Ganglionic Control of Movement in General (Classical Conception), and PD

Projections from midbrain (particularly, SNc) release DA at the level of the corpus striatum with contrary effects, mediated by two different, G-protein-linked DA receptor families located on spiny projection striatal neurons. Striatal projections to downstream sites (and cortical projections to striatum) have been understood to operate by way of so-called direct and indirect pathways. In an evolutionarily conserved way (4), the direct pathway inhibits, whereas the indirect pathway disinhibits, globus pallidus pars interna and the substantia nigra pars reticulata (GPi/SNr), which together are major inhibitory output nuclei from the basal ganglia. GPi/SNr output has distinct effects on thalamic nuclei to which they project, with ensuing inhibition (*via* indirect pathway) or disinhibition (*via* direct pathway) of thalamocortical drive (5). Additionally, in the normal state, DA, acting *via* distinct G-protein receptor subfamilies, facilitates direct pathway activity and inhibits indirect pathway activity. An additional “hyperdirect” pathway involves two excitatory projections in series, first from cortex to subthalamic nucleus (STN), then from STN to GPi/SNr, but the pathway may not be under DA influence (6).

The principal pathologic finding in PD is degeneration of DANs in the ventrolateral SNc (7). Striatal DA deficiency provides a rationale for use of the DA precursor, levodopa, as well as DA agonists in PD. As has been studied in animal models (selective SNc neuronal loss after exposure to 6-hydoxydopamine in rats or 1-methyl-4-phenyl-1,2,3,6-tetrahydropyridine in primates), DA depletion results in consistently observed alterations, including increased firing rates of STN, GPi, and SNr neurons (8). The classical conception maintains that the multisynaptic indirect pathway between corpus striatum and thalamic nuclei becomes overactive in the context of DAN degeneration. When levodopa- or agonist-associated complications arise (e.g., dyskinesia), ablation or deep-brain, high-frequency stimulation (DBS) of GPi or STN has been associated with convincing clinical benefits, including reduction in medication-associated dyskinesia and improvement in cardinal PD signs, including motor slowness.

The classical conception, despite its heuristic value, does not account for other known aspects of DA neurotransmission. As has been observed in early vertebrate species and mammals (4, 9), SNc neurons are activated by salient visual and other sensory stimuli. The influence of the environment on DAN firing has implicated DA neurotransmission in motor learning, in which action in the world is evaluated in an ongoing way. Specifically, DANs may code for deviations between predicted and real outcomes (10). A reasonable question arises as to how DANs track the time that passes from one event to another so as to determine a deviation or “error” from an anticipation or prediction.

## A DA Clock?

If an animal were trained to move to a target after a *self-timed* interval following a start-timing cue, then what neural mechanisms operate to determine the amount of time that precedes the movement to target? One can measure the time interval between a start cue and an ensuing movement objectively. But for self-timing to occur, some kind of subjective or internal time estimation must take place. SNc and DA may be involved in determining internal clock speed in time frames of seconds up to 2 min (2, 11–13).

**Figure 1:**
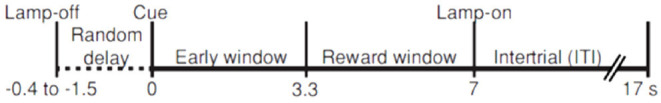
Hamilos et al. (14), with permission (Creative Commons Attribution License permits unrestricted use and redistribution provided that the original author and source are credited). Abbreviations are those used in the text. Schematic of self-timed movement task in Hamilos et al. (14). After a light cue, a mouse must wait 3.3 s before initiating a movement (a lick) for liquid reward. Termination of any single trial occurs at 7 s, although the intertrial interval (ITI) ends at 17 s.

**Figure 2:**
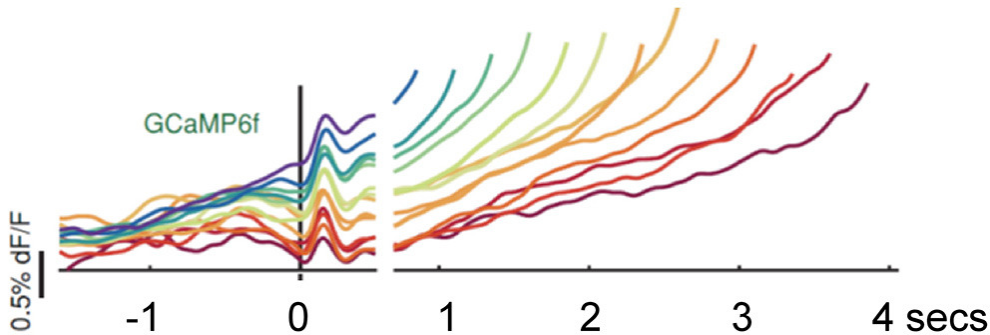
Hamilos et al. (14), with permission (Creative Commons Attribution License permits unrestricted use and redistribution provided that the original author and source are credited). Abbreviations are those used in the text. Average DAN GCaMP6f responses from 12 mice. The different colored traces correspond to averaged trial responses with different first-lick times after onset of a light cue. Averaged traces are aligned relative to both the start-timing cue (left of x-axis break) and timing of the first lick (right of x-axis break). The break in the x-axis indicates the change in plot alignment. Slope of the various colored traces correlates with time to first lick (steeper slope associated with earlier lick after 3.3 s; shallower slope associated with later lick after 3.3 s). dF/F: change in fluorescence intensity relative to resting fluorescence intensity.

Hamilos et al. (14) trained head-fixed mice to make self-timed movements to receive juice rewards (see Figure 1). Mice only received juice if they waited a prescribed interval (>3 s) before taking their first lick. After training, fiber photometry recorded the activity of SNc DANs expressing the calcium-sensitive fluorophore GCaMP6f. To control for optical artifacts, the authors simultaneously recorded co-expressed, calcium-insensitive fluorophores (“tdTomato” signals). DAN GCaMP6f fluorescence transiently increased after the start cue, then, during the self-timed interval, an up-slope or ramp-up of GCaMP6f fluorescence occurred, with a minimum time of onset of the ramp-up just after the cue-associated transient increase in firing. Slope of the ramp-up differed among trials in which mice moved early or late in relation to the cue. Differences in the steepness of ramping highly predicted the relative earliness or relative lateness of the first lick: steeply rising fluorescence preceded early licking; slowly rising fluorescence preceded later licking. TdTomato signals did not exhibit such ramping (see Figure 2).

DAN firing has effects on striatal neurons (dorsolateral striatum, DLS, in mice), as can be observed by simultaneous photometry of DLS. Optogenetic activation or inhibition of DANs, using light levels simulating physiological variations, resulted in contrary downstream striatal effects associated with different delays to movement within the time frame of a trial. DAN activation shifted the distribution of self-timed movements earlier (associated with a steeper slope of ramp-up); inhibition shifted distribution to a later time (associated with a shallower slope of ramp-up). The ramping signal unfolds over seconds and may precede first lick by as long as 10 s. Supra-physiological DAN activation caused large and sustained increases in DLS activity associated with immediate, non-purposeful body movements and disrupted task performance. An implication, corroborated by activations and inhibitions in the physiological range, is that DAN firing normally does not cause or suppress movement, but rather that DAN firing modulates a propensity to movement.

In the context of the experiments as described, propensity to movement relates either to the expectation of reward or, alternatively, to an ongoing evaluation of variability in that expectation, including situations in which reward prediction happens to be wrong. As has been described dating to the 1980's (10), DANs fire transiently in response to unpredicted rewards and to cues that correctly predict reward, but they pause when an expected reward does not occur. Only part of the work of an internal, neural clock is temporal measurement *per se*; another part may relate to the prediction of future salient events within very short, antecedent time frames.

An extensive time-perception literature (we focus on human studies in what follows) has specifically addressed short-term time perception in parkinsonism. How do the observations in mice, that DANs exhibit dynamic changes in firing over extremely short intervals, color our view of what DA deficiency entails for fleeting temporal perception in human disease?

## Clinical Literature Review

### Strategy

**Figure 3:**
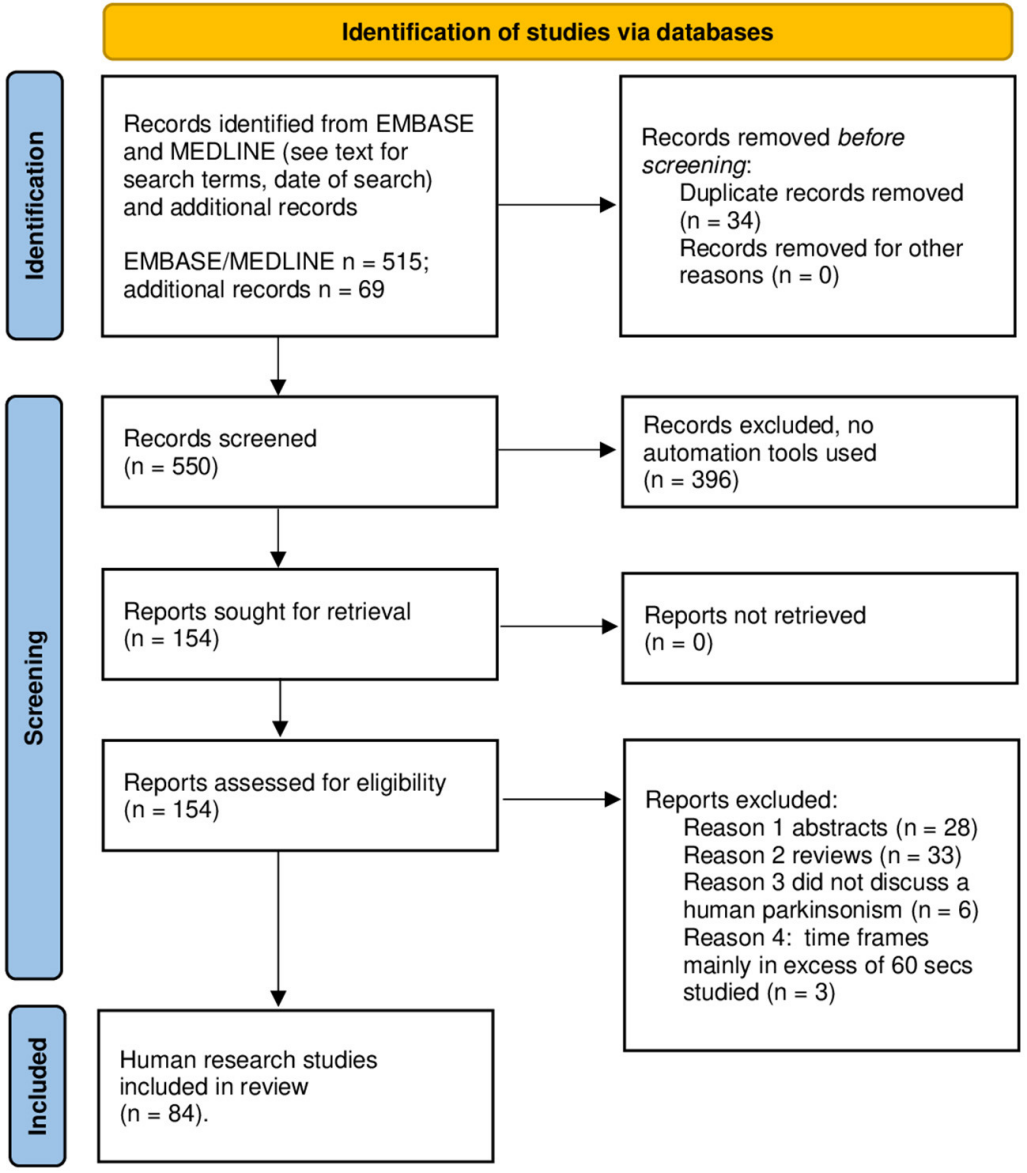
PRISMA 2020 template for the search and selection strategy used in this review. Review of literature according PRISMA 2020 flow sheet.

The literature search was conducted in accord with Preferred Reporting Items for Systematic Reviews and Meta-Analyses (PRISMA) guidelines. As of September 22, 2021, EMBASE and MEDLINE databases, interrogated with the search terms “temporal,” “perception,” and “Parkinson,” yielded 515 references. I added 69 papers selected from a PubMed search dating to 1971 and after review of bibliographies of seminal articles or reviews (1–3, 15–24). See Figure 3 for the results of the screening and selection process. A total of 84 studies in humans were studied; all were written in English. Supplementary Tables 1, 2 section provides details regarding all 84 investigations.

### Overview of Citations

#### General Comments

Clinical historical information—e.g., sidedness of onset (since PD typically presents asymmetrically), disease duration, degree of response to levodopa, clinical staging of parkinsonism and its severity in the OFF-anti-PD medication state (e.g., less or poor levodopa intervals during ongoing treatment, “OFF meds”) and the ON anti-PD medication state (e.g., good or better levodopa response intervals during ongoing treatment, “ON meds”) states, presence or absence of levodopa-associated dyskinesia, variation of dyskinesia in relation to dosing during a day, specifics of all PD medications and other concomitant non-PD drugs, medical comorbidities (e.g., subcortical white matter disease, illnesses other than PD, inter alia)—tends not to be detailed systematically or consistently in papers reviewed, although there is often an attempt to control for memory deficits in PD patients, as ascertained by various neuropsychological instruments. As has been discussed elsewhere (22), clinical variability likely contributes to disparities in reported data. In addition, although clinical assessments in the sample often used standard PD rating scales, there are inherent limitations in such instruments—e.g., a United Parkinson's Disease Rating Scale (UPDRS) score for bradykinesia may not reliably correlate with observed time perception disturbances (25).

#### Explicit vs. Implicit Timing Tasks

Coull and Nobre (26) and Avanzino et al. (27) offer a distinction that usefully, if only broadly organizes one's approach to the interval-timing literature. An *explicit* timing task requires that a subject provide a specific estimation of a time interval. How subjects report an interval varies among studies, as I will examine, but the gist of an explicit timing task is to provide an estimate of elapsed time. By comparison, an *implicit* timing task does not require a specific estimation of how long a stimulus or an action lasts. Yet, a subject *uses* timing information either to accomplish a task or to predict when something will occur in relationship to a prior event. Again, variations in implicit timing tasks vary paper by paper, but the gist of an implicit timing task relates to a process by which, for example, temporal expectations underpin the accomplishment of a task. A delay in temporal processing may explain variance from controls in intrinsic timing tasks.

A criticism which Coull and Nobre acknowledge, is that one cannot dismiss the possibility that implicit timing, especially across sub-second intervals, may contribute to accomplishing an explicit timing task. Yet, the extrinsic/intrinsic distinction allows one to examine any given study in relation to others of similar, basic methodology. Coull and Nobre add that in both explicit and implicit timing categories, performance may have either “motor” or “perceptual” features: e.g., the press of a button over some duration (a motor aspect of an explicit task); judgment of a longer vs. shorter interval in relationship to another interval (a perceptual aspect of an explicit task); an action based on memory of a preceding time interval (a motor aspect of an implicit task); or, a prediction or expectation within a stipulated time frame (a perceptual aspect of an implicit task). In what follows, I address papers according to the explicit-implicit distinction, although each study in either category has its own nuances (for details per study, see Supplementary Tables 1, 2). With respect to anticipation resulting from an implicit temporal estimation, as I discuss below, a very different literature review than the current one would be necessary, since a number of studies—many of which enlist functional brain imaging—finds that prediction and expectation involve more areas of brain than those under direct DA influence.

#### Explicit Timing: Estimates of Time Duration

Consistent with earlier work (15–18, 28–33), Malapani et al. (17) studied OFF meds vs. ON meds states [see also (34)]. A peak-interval timing procedure was their index of time estimation. As described both by Malapani et al. (17) and by Matell and Meck (35), peak-interval timing involves a subject watching a series of images on a screen while remaining “aware” of the amount of time an image remains on the screen [e.g., 8 vs. 21 s (secs)]; then the subject is instructed that an image will appear for either 8 or 21 s. The subject is asked to press a button based on when she expects that the image will disappear. She is not supposed to subdivide duration in any fashion (no counting to herself, for example). During training, the subject is told for just a fraction of the trials that the button was pushed early, late, or accurately. After training, accuracy (button press at 8 or 21 s) and variability around either peak time are studied. Based on extensive experience in animals and humans, accuracy is often quite reasonable, but variability increases proportionally to the duration being timed (a scalar property). Systematic under- or overestimation of time tends to self-correct with training, but not all studies describe the scalar property (36).

Malapani et al. (17) reported that patients OFF meds show significantly impaired accuracy for both the 8- and 21-s peaks, with a tendency to err on the late side after 8 s and to err on the early side before 21 s. In addition, also in the OFF meds state, the variability around peaks was greater around the 8-s peak than around the 21-s peak—a non-scalar variability. Such changes were not observed either in the ON-meds state or in controls. Corroborating Pastor et al. (33), ON states differed from OFF states in terms of PD patients' time estimations. Malapani et al. concluded that dopaminergic deregulation—specifically, the OFF state—is associated with distortion of accuracy of time estimation, greater variance around peak times, and loss of scalar property. All three aspects have bearing on the subject of explicit timing tasks, perhaps especially their non-scalar property (34, 36–38), although not all studies agree that the three observations hold true in PD or other parkinsonisms [for counterpoints, see (39–41)].

Applying a explicit timing protocol used in their experience with transcranial magnetic stimulation (42, 43) noted time estimation migrations [as described by Malapani et al., (17)]–i.e., overestimation of 5-s intervals and underestimation of 15-s intervals–in PD patients OFF meds/OFF deep-brain stimulation (DBS) of subthalamic nuclei (STN) vs. the same patients OFF meds/ON DBS of STN, as well as ON meds/OFF DBS of STN. Other DBS experience suggests that effects on time duration estimation vary in relationship to the frequency of stimulation delivered to STN (44). With PD treatment (either ON meds or ON DBS of STN), time estimations improved, but did not fully correct (43, 45). Merchant et al. (37) reported two subgroups of PD OFF meds patients—one group with highly variable and the other with variability near control values in time estimation; variances improved in the ON-meds state.

Errors in the determination of duration have been thought to point to a dysfunction of a hypothetical internal clock operative in the millisecond (msec) range, as many groups have attested (15, 16, 28, 46–50). The hypothetical clock may err in non-PD contexts. In an interesting comparison of young, normal controls, elderly controls, PD patients OFF meds, as well as presymptomatic and symptomatic patients with Huntington's disease, time estimations erred (both too fast and too slow) among *all* cohorts except the young controls (51). Fielding et al. (52) also observed that visual saccade generation in cued and uncued contexts was impaired in both PD and Huntington's disease.

#### Explicit Timing: Estimation, Production, Reproduction, and Variations

The tests used to quantify subjective estimates of duration vary methodologically (22). In time *estimation* tasks, an interval is presented, then a subject is asked to estimate its length to the nearest second (verbally). In time *production* tasks, a subject is asked to perform some movement (e.g., pressing a button at start and end) for period of time that they are asked to produce (e.g., 30, 60, 120 s). In time *reproduction* tasks, a subject is presented a target interval visually or aurally, then, later, they are asked to perform some movement (e.g., sustained key press) for the amount of time that the target interval had been presented. The peak-interval timing procedure used by Malapani et al., (17) is a kind of time reproduction task. Time production and reproduction tests have been performed concomitantly or in variations of either (34, 39, 53, 54). In addition, there are *bisection* and *trisection* tasks: in a bisection, a subject is asked to learn two durations (e.g., a short and long one; a trisection involves three durations), then to judge whether another, new duration—one that lasts somewhere between the short and long—is closer to the short or to the long interval (55). What one learns from such subtly different tests has been debated (56, 57), and the various methods used across studies may contribute to their mixed or sometimes contradictory results.

Allman and Meck (38) offer a different cautionary note. The ON-meds state in PD has not been uniformly associated with improvement in temporal perception tasks compared to the OFF-meds state (31). In the mid-1990's, with the advent of the treatment of PD with DBS, especially of STN (pallidal DBS has not been studied in depth with respect to timing), additional subtleties have been observed (58), as we discuss, below. In some reports, PD patients (both ON and OFF meds) exhibited nearly normal timing-task performance (29, 41, 59). There has even been contrary results from the same research group: Perbal et al. and Perbal-Hatif (49, 50) reported migration effects similar to Malapani et al. (17), but Pouthas and Perbal (39) corroborated Malapani et al. only for time production, not reproduction tasks. Other investigations have examined short-term time estimations when different time frames were examined in one as opposed to separate session/s (54), in non-PD movement disorders (52, 60, 61), and in association with event-related-potential or electroencephalographic parameters (62–66).

Perhaps as a consequence of contrary data using diverse methodologies, investigators have considered other context dependencies—e.g., study of auditory or visual stimuli in time estimations ON meds (55, 67); of timing cues during gait both ON and OFF meds (68); or of time production *vs*. reproduction tasks both ON and OFF meds (34) or both ON and OFF meds or ON and OFF DBS of STN (45).

Using a trisection method in which subjects needed to decide whether time intervals were short (200 ms), medium (550 ms), or long (900 ms), Zhang et al. (69) found that PD subjects had difficulties in discriminating short- and medium-time epochs, but, interestingly, they exhibited impulsive decision strategies that appeared to bias them toward premature responses. In a temporal bisection task, Mioni et al. (70, 71) asked PD patients to “memorize” 400- and 1,600-ms intervals, then later to judge whether a new stimulus was closer to 400 ms or to 1,600 ms (the stimuli lasted 400, 600, 800, 1,000, 1,200, 1,400, and 1,600 ms, and were presented randomly). A so-called Weber ratio (WR) quantified variability in responses, PD subjects tended to underestimate time and to exhibit widely distributed individual WR's, the latter suggesting lower sensitivity to time estimation generally. Reminiscent of Malapani et al. (17), Terao et al. (72) found that time reproduction of very short time durations was longer in PD subjects [and even longer in Progressive Supranuclear Palsy (PSP) subjects] and shorter for long-time durations (even shorter in PSP). Terao et al.'s methodology included various tests in concert with time reproduction tasks.

Temporal misperception, as opposed to general cognition measured by neuropsychological testing, may be subtle in its manifestations, and may not be rectified with anti-PD treatments. Bernardinis et al. (73) found anomalous time estimations, especially in long rather than sub-sec time frames, that did not improve with either meds or DBS of STN [see also (45, 58)]; and varying DBS parameters had no effect on duration- and beat-based timing (74). Alternatively, Wojtecki et al. (44) observed improvements in time production and reproduction associated with high-, but not low-frequency STN of DBS. Honma et al. (75) argue that stopwatch training even in medicated PD patients can improve time production tasks as well as performance in go-no-go tasks (associated with decreased impulsivity).

Diverse observations notwithstanding, is DA deficiency itself responsible for any timing disturbance? In correlation with quantified striatal DA transporter (DaT) deficits, studying both time production (33, 53) and time reproduction (17), Honma et al. (57) found that, 0–5 s after a cue, time production overestimated, then began to underestimate time. The underestimation continued to increase from 10 to 300 s. In time reproduction, PD patients overestimated time in the first 2 s, then normalized in comparison to controls. Striatal DaT deficits correlated with underestimation beyond 10 s in reproduction tasks.

#### Implicit Timing: Processing Delay or Other Aberrancy in Processing

Various groups (16, 28–30, 41, 68, 76) have scrutinized the relationship in PD between a cue (e.g., a visual prompt) and cued action (a reaction based on the prompt, but after a delay period). De Lancy Horne (76) studied a population of PD patients who had undergone thalamotomy, a procedure associated with improvement in tremor and rigidity in PD. A mean-square root of reaction times reduced significant variance in the data, but, overall, PD patients exhibited slower reaction times in two of three tasks compared to age-matched controls, but different lengths of delay period (all ≥10 s) did not affect the slowness of reaction times. By comparison, another early paper examining non-linear components of visual evoked potentials (VEPs) found differences between controls and both untreated and treated PD when examining phase-shifting VEP components presented simultaneously, suggesting that processing of visual information is disturbed in PD (77). Investigators (31, 78) have also examined various stimulus-to test (cue-to-cued-response intervals). During those intervals, Sagar et al. (78) introduced other stimuli. Subjects with PD showed disproportionate deficits in content recognition—viz., in the actual content of a recollection—only at the shorted stimulus-to-test intervals, in comparison to subjects with Alzheimer's disease whose deficits manifested at all stimulus-to-test intervals.

Rammsayer and Classen (79, 80) studied information processing in PD over msec time frames by way of subjective estimations of durations of stimuli: impaired processing in those very short times, they argued, was a trait marker of decreased DA activity rather than a state marker of clinical symptomatology. The rationale behind examining activity inside 500 msecs dates to a contention from the late 19th century that, across extremely short time intervals, information processing is not mediated cortically, but rather by way of subcortical structures (80, 81). Length of scrutinized time frames varies in the early literature (15, 32); Lange et al. (53) asked patients to judge time intervals of 10, 30, and 60 s—up to two orders of magnitude greater than the interval studied by Rammsayer and Classen.

Riesen and Schnider (40) studied short temporal processing (~1 s) in patients ON meds (various daily doses among subjects) by asking them to determine the longer duration of sequentially presented images. The authors determined that, for PD patients, two stimuli must be separated by a longer interval compared to controls in order for them to perceive events as separate. In Shipley et al. (82), using a method that attempted to determine the minimum stimulus duration to discern an order embedded in simple visual stimuli (viz., a sequence of letters; images displayed from 100 to 700 msecs), PD patients ON meds exhibited, as in Riesen and Schnider, a difficulty in monitoring and distinguishing temporally distinct events in the short term. Johnson et al. (83) focused on what they called inspection time in PD (both ON and OFF meds—viz., inspection of graphical variations of the Greek letter *pi*). They found that in both the ON- and OFF-meds states, PD patients required longer inspection times by about a third compared to controls, although the overall inspection times for both PD groups and controls were <200 ms.

In a study of “foreperiods” before cued involuntary and voluntary motor reactions, Jurkowski et al. (84) found that for older persons without PD and for those with PD OFF meds compared to young controls, foreperiod time had very little effect on voluntary reaction time (which was delayed in response to all antecedent foreperiods, 1.0 s, 2.5 s, 4 s, 6.5 s, in both the elderly and in PD). When involuntary reaction was studied (latency to eye blink in response to a puff of air), the short (1 s) and longest foreperiod (6 s) each was associated with a quicker eyeblink (compared to the 2.5-s and 4-s foreperiods), although latencies were nevertheless longer in the elderly and in PD patients when compared to controls. As Bloxham et al. (31) had speculated years before, in general, patients with PD—perhaps the elderly as well—perform as if they were carrying out another task at the same time, but they do not dual task efficiently [for a contrary, contemporary opinion see (85, 86)].

A foreperiod methodology has been used as well by Tomassini et al. (87) who employed Bayesian analysis to observe that DA deficiency was associated with increased subjective uncertainty about predictions in time. Zokaei et al. (88), also using a variation on foreperiod, introduced a temporal orienting cue to help divert attention from distractors; they found that the cue did not help PD subjects OFF meds, but did help ON meds. The authors concluded that the benefits of cueing may relate to specific processing demands of a task.

Altered integrations, in PD, have been variously reported between sensory input [including a study with distractor stimuli (89)], motor performance, and memory or other cognitive tasks [(58, 60, 62, 68, 90–105), see (64, 65, 106) for reviews; Table 1 summarizes these particular studies]. In the spirit of context-dependent studies in *explicit* timing tasks (described above), PD may increase the perceived duration not of time, but of an action [(45), but see (105) for a contrary view]; may corroborate a migration effect (overestimation of short intervals, underestimation of long ones), especially when an emotionally salient event is being timed (71); may not increase the time of anticipatory eye movements (98); may shorten latency to acoustic startle (96); may prolong the perception of syntax in a piece of music (91); may prolong time spent in the appreciation of works of art (107); and may alter the subjective rating of an aural rhythm's complexity, depending on beat frequency (84).

**Table 1:** Representative implicit timing studies regarding altered somatomotor processing (all vs. controls, with other intergroup analyses, as indicated).

**References**	**Method(s)**	**Clinical context(s)**	**Duration(s) examined**	**Summary observations**
Guehl et al. (58)	Auditory processing study, ascertainment of duration of gaps in continuous noise, but a second test in which in which continuity of noise varied between trials, and study of what happens at the moment the continuity changes	OFF meds and DBS of STN, then ON meds, then ON STN of DBS	Variable gap duration, starting at 50 ms	Deficit in detection of gaps in PD, with incomplete rectification with STN of DBS (no rectification with meds); but, in second test, no difference between PD and controls
Beudel et al. (60)	Predictions regarding a moving target in PD and degenerative cerebellar disease	ON meds for PD group	1 s presentation time	Impaired velocity estimation of a moving target in PD; prediction of moving target's terminus in space impaired in cerebellar disease
Friederici et al. (62)	Event related potential (ERP) study, correct vs. incorrect sentences presented aurally	ON meds	P600 peak at ~0.6–1.0 s	Impaired late (P600) processing, preserved early processing in PD
Almeida et al. (68)	Timing cues in control of gait	ON and OFF meds	60, 80, 100 steps/min	PD patients either ON or OFF with “locked” step length regardless of external cues
Beudel et al. (90)	Threshold for detecting change in velocity of a moving image	~4 h after last dose	Threshold for perceived velocity change ~75 msecs in PD and controls	Unchanged velocity perceived as acceleration (acceleration bias) less in PD, related to degree of bradykinesia
Bellinger et al. (91)	Threshold study while listening to original, then altered music	ON meds	Delay time interval 80–300 ms	No difference in “just noticeable difference,” but impaired detection of 220–300 ms intervals
Conte et al. (92)	Somatosensory temporal discrimination with kinematic analysis	ON and OFF meds	Interstimulus intervals in 10- ms steps	PD: abnormalities in the temporal coupling between tactile information and motor outflow
Jahanshahi et al. (93)	Synchronization and continuation motor tasks to auditory cues, positron emission tomography (PET) study	ON (apomorphine) and OFF meds	50 msecs per tone, interstimulus interval of 1 s	OFF meds: cerebellar activation more prominent, less frontal activation
Lee et al. (94)	Visuospatial memory task	OFF meds	500 ms memory array	PD: impaired filtering of distracting aspects of an array image
Husárová et al. (95)	Predictive motor timing tasks	OFF and ON meds, but 50% drug naive	Button push every 2.5–4.5 s	PD: “trouble postponing” action until the proper moment
Carlsen et al. (96)	Acoustic startle stimulus vs. “go” stimulus	ON and OFF meds	“Premotor” reaction time (RT) ~173 ms	Shortening of premotor RT by startle whether ON or OFF meds
Miller et al. (97)	Synchronization to tone sequences; positron emission correlation	ON meds, then placebo pill	Tones separated by 500, 1,000, 1,500 ms	*Better* synchronization accuracy with *greater* striatal DA denervation
De Hemptinne et al. (98)	Visual smooth pursuit tracking task in forward direction then in reverse	early PD, most still drug naive	Target moves for either 1,200 or 2,400 ms	Anticipatory eye movements less frequent in PD, but timing of anticipation matched controls
Bieńkiewicz et al. (99)	Synchronization of movement to a beat	ON meds	Intervals between sounds 1.5 or 2.5 s	Poor synchronization related to severity of PD, not to spatial constraints of the task
Feher da Silva et al. (100)	Temporal order judgment (TOJ, subjects asked which of two images appears first)	ON meds	Variable interval depending on correct responses, beginning at 117 ms	TOJ impaired in PD and in healthy elders
Biswas et al. (101)	Beat or rhythm discrimination	ON/OFF not specified	4 tests used, different beat intervals	PD: association between impaired perception of rhythm and tests of verbal working memory and focused attention
Breska et al. (102)	Perception of rhythmic regularity vs. remembered temporal associations	ON meds, comparison group with cerebellar ataxia	Target intervals of 600 and 900 ms	PD: impairment in rhythm-based temporal predictions. Cerebellar disease: impairment in interval-based temporal predictions.
Nelson et al. (103)	Vibrotactile stimulation, inhibition of effect of transcranial magnetic stimulation (TMS); functional imaging study	ON and OFF meds	Long latency afferent inhibition (LAI), duration of sensory stimulation 200–1,000 ms	Reduced LAI ON and OFF meds; deficient activation of contralateral primary sensory cortex and reduction in sensorimotor integration (less effect of TMS over primary motor cortex, suggesting deficient somatosensory processing)
Yabe et al. (104)	Perceived timing of a past event intended to trigger action	ON and OFF meds	Random delay to trigger, 1–2 s	PD: sensory event triggering an action perceived to have occurred earlier in time
Cao et al. (105)	Study of movement perception; perceived temporal durations of a visual presentation in two parts–e.g., upright vs. inverted	OFF meds	Presentation durations of a second part, ranging from −900 ms to +900 ms, compared to 1- s first presentation	“Temporal dilation” (e.g., tendency to perceive an upright motion as lasting longer) reduced in PD

Degos et al. (108) studied visual saccades initiated after variable foreperiods before movement in PD (both ON and OFF meds) and PARKIN-associated parkinsonism (the PARKIN gene, also known as PRKN, encodes an ubiquitin ligase; various mutations of the gene have been associated with an autosomal recessive parkinsonism, typically of juvenile onset). Latency to saccades decreased with increased foreperiod durations for all groups, both PD patients ON and OFF meds and controls, after controlling for motor reaction time. The implication is that implicit monitoring of elapsed time appears to be functional in PD. Yet, a within-group analysis found that the influence of an antecedent (“n-minus-1”) foreperiod was evident among controls and PARKIN-associated parkinsonians, but not among PD subjects both ON and OFF meds: what would appear to be an impairment in short-term memory for a previous foreperiod could not be attributed, in the authors' view, to a general slowness of processing if the immediately preceding foreperiod effect for an oculomotor task did not materially differ between any of the groups.

#### Implicit Timing: Perception of Movements in Time

An elegant study by Fiorio et al. (109) studied unilateral bradykinetic-rigid PD (OFF meds) neurophysiologically. They induced index finger abduction or wrist flexion by stimulation of the motor point of the first dorsal interosseus muscle (FDI) or flexor carpi radialis (FCR). Subjects, all blindfolded, were asked to report whether pairs of stimuli separated by various time intervals resulted in finger abduction (action of FDI), wrist flexion (action of FCR), or both. The shortest time interval at which subjects reported two discrete movements in time was called the temporal discrimination movement threshold (TDMT), specific to the proprioceptive domain. Comparisons were made between the affected and unaffected sides in PD patients and in PD patients vs. controls. Mean TDMT was higher in the affected than the unaffected arm (111 ms and 95 ms, respectively); PD patients had higher TDMT's than controls only on the affected side (111 ms and 88 ms, respectively). Lyoo et al. (110) used a different TDMT protocol, but observed that, among 30 *de novo* cases of multiple system atrophy, an atypical parkinsonism with subtypes (e.g., akinetic-rigid, ataxic), significantly increased thresholds correlated with bradykinesia ratings. TDMT has also been studied in asymptomatic and symptomatic PINK 1 heterozygotes in a comparison with symptomatic homozygotes (the PINK 1 gene encodes a mitochondrial protein kinase and has been linked to autosomal recessive parkinsonism): when compared to controls and asymptomatic heterozygotes, all symptomatic cohorts demonstrated higher TDMTs (111). Lee et al. (112) corroborate TDMT abnormalities, especially in OFF-state gait freezing, a particularly bothersome and dramatic PD phenomenon.

Anti-PD treatment targeting motor deficits may or may not correct observed implicit timing alterations compared to controls. When compared ON meds and OFF meds, PD subjects may not exhibit improved threshold discrimination (59). Consider, by comparison, the seeming contradiction that medications improved discrimination thresholds, but DBS of STN may not (83, 113).

Nevertheless, temporal discrimination thresholds have been examined in various ways and at different body locations in PD (25, 73, 90–92, 113–117). PD patients as well as subjects with non-PD movement disorders may exhibit implicit timing alterations (50, 51, 61, 116). Such data provide support for the idea that a movement disorder may involve higher-order *sensory* dysfunction (117).

#### A Comment on Predictive Timing

A question arises in light of the implicit timing literature whether predictions of future events are a function of implicit timing, explicit timing, or a combination of both. Ivry and Keele (32, 102), as corroborated by other studies [e.g., (118–120)], suggest that prediction of events likely involves more than a single neural mechanism—and not all those mechanisms or networks may be under direct DA influence. For example, past temporal regularity may allow a person to anticipate a future event, but, if such regularity is not present or is not perceived, then neuropsychological study (102), functional imaging and electrophysiological studies [some touched upon in this review, including (26, 27, 62, 64–66, 93, 114)] suggest that multiple cortical and subcortical domains may be involved, depending on specific context. Breska and Ivry (102) summarize neuroimaging studies that highlight the role of cortical (e.g., left inferior parietal lobe, supplementary motor area, and right inferior frontal cortex) and cerebellar nodes—striatal locales to a lesser degree—in experimental paradigms that attempt to reproduce prediction in time. This review does not significantly broach that expanding literature, although consideration of DANs in time keeping and prediction is not therefore obviated.

## Discussion

The subjective estimation of time intervals (either in explicit or implicit timing tasks) has been studied extensively in PD. The studies included in this review are not at all univocal in their conclusions, but they altogether point to an open question, relevant both to basic and clinical science: why might DA play a role—or why does an otherwise clinically significant deficiency in DA play a role—in timekeeping at all?

In response, consider DANs and the determination of inter-event durations in the context of expected reward. As Hamilos et al. (14) observe, DANs fire in steep or gradual ramped fashions that predict, respectively, when earlier or later movement will occur. What information can be said to be communicated by different slopes of firing—or, put differently, what do slopes *inform* about the context in which the animal finds itself at any given time? DANs do not trigger movement *per se*, as was corroborated when DANs were optogenetically activated at physiological levels. A first-pass resemblance to interval timing in which a clock system *senses* duration in the world, then inputs to an “accumulator” (a kind of working memory), as has been studied in the Pavlovian conditional response (121), suggests that DAN ramping relates to the probability of response in specific contexts or circumstances.

Only a minority of SNc neurons can be visualized at a time in a photometric study of SNc in mice, but it is plausible that the extreme loss of DANs in clinically manifest PD (7) may result in a dys-calibration of accumulator function. The consequence would not necessarily be an inability to move, but rather a skewed probability function that could manifest variously–either as slowness, impulsivity, perseveration, or some combination of all three features. In clinical PD phenomenology, slowness does in fact mix with other aspects, as one observes in a festinating gait, which combines both fast and slow aspects. Likewise, in gait freezing, the stance is not a fixed motionlessness in space; it is often apparent that a PD patient is trying to step forward, though the perseverative effort is both fitful and unsuccessful.

With respect to the provocative, but not universally accepted observation that PD patients overestimate very short time intervals and underestimate longer ones, some background discussion is in order. Karl Vierordt's *The Experimental Study of the Time Sense*, published in German in 1868 (122), introduced a psychological “law” in which, for example (based on study of himself, but corroborated by other data reported in the book), durations < ~2.5 s are overestimated and those longer than ~2.5 s are underestimated *normally*, with an “indifference” point (at ~2.5 s), when estimation of duration is transiently veridical. The validity of Vierordt's law, instances of its violation, and the variability of the indifference point (depending on durations studied, extending to many minutes) have been reviewed (123), and iterative Bayesian analysis has been applied to some of the original 1868 data to show that the law's validity varies with testing protocols, especially when durations are randomly ordered (124). Variation in manner of study certainly characterizes the studies included in this literature review. One should not conclude that the variability forces one to dismiss the idea of a temporal-sensory disturbance, since there are interesting suggestions that time misperception may underpin not only PD, but also other movement disorders (e.g., Huntington's disease and dystonia), as somatosensory threshold data and many other, though by no means all, studies that address implicit timing tasks have demonstrated, perhaps more consistently than in studies of explicit timing.

The goal of treatment in a parkinsonism is to allow for the greatest possible functional benefit in real time. The adjective “real” is essential: time judgments in life are not completely veridical compared to an objective clock, and, in real life, desires, plans, goals, and past experience (in both short and sometimes very long time frames) color both moment-to-moment and overall survival.

Recent data suggest that primates use Bayesian strategies by which preceding events alter probabilities of later ones in a trial-by-trial, ongoing fashion, all as a function of cortical representations of elapsed time (125). At the core of such a strategy is an incremental procedure in which differences in temporally successive predictions drive learning, as opposed to decisions made on the basis of past prediction errors of temporally isolated events (126). Mikhael and Gershman (24) have reviewed dominant models in the DA-and-timing literature—Table 2 provides a summary based on their paper and this review. They usefully observe that all modeling contends with minimizing differences between true and estimated values (e.g., of time; of a reward in time, associated with a “reward prediction error”) as a function of different *gradients* (a term also used in 127) that represent, as in any ongoing analysis, the effect of antecedents on future judgments of difference between the estimated and the true. It may not be the case that successive trials necessarily lead to a zero difference between truth and estimation, and, more probably, a zero difference never happens, neither absolutely nor in perpetuity. It should not surprise that, in real life, judgments can err in either direction, both toward the veridical or very far from it (but bidirectional updating continues, by definition). Mikhael and Gershman (24) further observe that modulation of timekeeping mediated by DA would have an effect on learning in the above iterative sense, especially in contexts that are either highly complex (“noisy”) or stochastic (random).

**Table 2:** Summary of theories in DA-and-timing literature.

**References**	**Type of modeling**	**Brief description**
Killeen and Fetterman (127)	“Sequential state” (related to pacemaker- accumulator), behavioral theory of timing	Transitions between behaviors a function of pulses of an internal clock; cause of a behavior in state *n +1* is the reception of a pulse while a subject was engaged in a behavior corresponding to state *n*. The transitions in behaviors follow a Poisson distribution. The authors do not discuss subjective scales of time perception.
Gibbon et al. (2)	Pacemaker- accumulator	Striato-thalamo-cortical loops critical for mnemonic encoding of comparison intervals in timing tasks; DA increases scalar variability, increases clock speed.
Machado, (128)	Pacemaker- accumulator; learning theory of timing	Reinforcement a critical component of temporal regulation (an early example of using reinforcement learning in the perception of time intervals).
Matell and Meck (35), Allman and Meck, (38), Meck et al. (64)	Striatal beat frequency	Interval timing based on coincidence detection of oscillatory processes in cortico-striatal circuits; at the onset of a to-be-timed signal, a distributed network “resets” its oscillation; DA involved in the reset.
Fortin, (129)	Attentional control of timekeeping	Different DA effects in terms of directing or diverting attention to timekeeping as a function of a task.
Shea-Brown et al. (36)	Firing rate model of parkinsonian deficits	Model motivated in part to explain non-scalar aspects of the migration effect [see discussion of Malapani et al. (17) in the text], based on an idealized recurrent, excitatory neural network; trial-to- trial variations outside the scope of the model.
Simen et al. (130)	“Stochastic ramp and trigger” model	Based on a diffusion (as in Brownian motion) modeling, “random walk” analysis in infinitesimal time steps) of neuronal spike trains, an attempt to account for temporal integration, response thresholds, variable clock speed, as well as resetting and learning during intertrial intervals.
Avanzino et al. (27)	Neural network (study of several neurological diseases other than PD)	Lateral cerebellum, basal ganglia, sensorimotor and prefrontal cortex together constitute an internal clock; attempt to differentiate two types of timing: *explicit* (e.g., movement over a duration) and *implicit* (e.g., coordination of individual movement in relationship to the external world).
Teixeira et al. (131)	Time estimation training	Hypothesis that time estimation tasks, as opposed to their use in experimental measurement, could be used in neural adaptation and rehabilitation, specifically in PD.
Meck et al. (13), Luzardo et al. (132), Ratcliff and McKoon (133)	Drift diffusion model, Resorcla Wagner (RW) associative model	“Drift” refers to rate of accumulation of data. RW was a model to account for a blocking effect in which a novel conditioned stimulus does not become associated with an unconditioned stimulus if it is reinforced only in relation to a prior conditioned stimulus. An attempt to account for the timing of a conditioned response.
Schultz et al. (10), Mikhael and Gershman (24)	Reward prediction error	DA a teaching signal that allows basal ganglia to predict future rewards in reinforcement learning tasks; see text for further discussion.

An important therapeutic implication is that current therapy to spare DA, or to enhance DA agonism, or the long-term, high-frequency stimulation of non-SNc sites may simply not rectify the inherently dynamic capacity of DANs in the pre-disease state. Loss of temporal dynamism has wide ranging network effects. If we consider that A8, A9, and A10 DA cell groups not only project to striatum, but also receive afferents from diverse locations, including the striatum itself (see Table 2 for a summary), DA denervation in PD represents both and output and input problem: not only is there loss of DA innervation of relevant pathways to striatum, then to downstream sites, but also the role of DANs in afferent integration is compromised—the latter may be associated with time misperceptions in movement disorders not themselves associated with selective DAN degeneration.

A greater probabilistic understanding of how pre-planned or spontaneous movement begins to transpire across very short time frames may inform new, more exquisitely variable, and highly time-sensitive deliveries of treatment. For example, as reviewed by Jenkinson and Brown (134) and corroborated by Hamilos et al. (14) in their data presented above, increases in striatal DA happen at 500–600 msecs after DAN discharges that constitute a phasic burst. In association with phasic DAN discharges, striatal interneuronal oscillations the beta range [13–30 hertz (Hz)] dampen in an intermittent or “scalloped” fashion. In PD, reduced beta synchrony may be a marker for motor impairment. It may further be the case that there are different ranges of beta oscillations—e.g., low beta (with mean frequency of ~15 Hz) and high beta (mean frequency of ~26 Hz)—with the most robust motor improvements in the ON state associated with dampened low-beta, but not high-beta oscillation (135). Dating to early work on synchronization of neural activity [reviewed in (136); see (66) for a discussion of electroencephalographic synchronization specifically in a timing task in PD], temporal patterns have been understood as a repository of information useful in understanding both normal physiology and pathophysiology: temporal coding, based on patterns observed in short-me frames, along with their distributed effects (e.g., striatal beta oscillations), could lead to refined ideas about what specifically changes in an anti-PD treatment (e.g., dampening specifically of low-beta oscillation) and the relation between a treatment-related change and other temporal coding associations (e.g., coupling between beta and other frequency oscillations in different time epochs or across different anatomical locations).

I acknowledge obvious limitations in my review. To concentrate on the role of DA in subjective time perception is not to say that DANs are the only timekeepers in the central nervous system, nor do DANs exclusively clock time. Inspecting the various theories posed in the DA-timing literature (Table 3), as Cruz and Paton (145) have opined in a different context, one notes that DA signaling must be heterogeneous (it does not merely keep time). Further, DA effects must be spatiotemporally ramified if any one of the theories is even partly valid. Transitions in behavior, mnemonic encoding, reinforcement learning, conditioned and unconditioned response, and ongoing correlations between explicit and implicit perception of the world are functions that intuitively should involve much of the brain as one gleans from even a cursory inspection of the *predictive* timing literature.

**Table 3:** Afferents and efferents to midbrain DA cell groups, references in parentheses.

**DA cell group**	**Anatomic location of DA cell group(s)** **(137, 138)**	**Summary of afferents**	**Summary of efferents**	**Comments** **relevance for moment-to-moment motor control**
A8, A9	A8: ventral lateral midbrain tegmentum, roughly at the axial level of red nucleus and inferior colliculus; A8 merges with A9; A9 comprises **substantia nigra pars compacta, SNc** Alternative organization: **DORSAL AND VENTRAL** tiers (both tiers composed of A8 and A9 cells groups)	Striatum (striosomal compartment), cortex, habenula, optic tectum/superior colliculus, pedunculopontine tegmental nucleus (PPTg, cholinergic and glutamatergic afferent) (4); rostromedial tegmental nucleus (RMTg; gamma- aminobutryric acid, GABA, afferent) (139)	SNc contains not only neurons that project to striatum, but also to limbic and other neocortex (138)	1.Striosomal compartment (pale staining areas in striatum) project ***back to*** SNc (140) 2. (lateral) habenula projects to SNc and VTA (141); afferent firing associated with averse events (results in ↓DAN firing) 3. midbrain reticular formation (location of RMTg) also receives input from the extended amygdala
A10	Unpaired midline collection ventral to red nucleus, bounded ventrally by interpeduncular nucleus; general area termed **ventral tegmental area, VTA**	As observed by rabies-mediated transsynaptic tracing (142): bed nucleus of stria terminalis, central and extended amygdala, deep cerebellar nuclei, dorsal raphe, caudatoputamen, internal globus pallidus, laterodorsal tegmentum at pontomesencephali c junction (see PPTg, above), habenula, nucleus accumbens, parabrachial nucleus, preoptic area, paraventricular hypothalamic nucleus, ventral pallidum, zona incerta.	VTA neurons project to nucleus accumbens and ventromedial caudate-putamen and broadly across primate neocortex (137); also to bilateral locus ceruleus and lateral parabrachial nuclei (142). Beier et al. (142) comment that a top- down, anterior cortex- to-VTA-to-nucleus accumbens circuit is reinforcing.	Early literature (143) discusses conjoint role of cortical/glutamatergic, STN/glutamatergic, ventral pallidal, and PPTg afferents on DAN firing patterns in both SNc and VTA. DA release from VTA neurons regulated by GABA transmission from ventral pallidum (tonic inhibition, influence on tonic “population activity” in VTA) and by cholinergic and glutamatergic transmission from PPTg (influence on burst firing) (144).

The subjective perception of time simply does not “belong” to a specific brain region (146); among the papers in this review, even in the context of DAN loss in PD, other subcortical structures (perhaps cerebellum in particular) may intervene to compensate for a DA-related timing deficit (80). Pertinent, however, to DANs and striatum in particular, Cruz and Paton (145) editorialize on recent work (147) that leverages high-resolution visualization of signaling directed to the richly DA-innervated striatum, including its striatal sensorimotor and associative or limbic domains: Hamid et al. (147) describe “wave-like spatiotemporal activity” across striatum, with DA transients organizing into local striatal clusters, which appear to be tailored to task demands. Of interest in relationship to the mouse data presented at the start of this review, DA *ramped* signaling may pass to striatal *sub*regions in relation to demands of a task in real time. DA dynamism is temporally varied in very short time frames at the level of SNc and, downstream, it exhibits elegant and intricate spatial ramification to accomplish intended action.

## Conclusions

Animal study suggests a role for DA in timing related to planned movement. The subjective perception of time informs the bradykinesia or slowness that characterizes PD. Loss of dynamic variability in DA neuronal activity in very short time frames may be fundamental to the pathophysiology of parkinsonism and perhaps other disorders manifesting incorrectly timed coordination or sequencing of purposeful movements. In a literature review spanning 40 years, clinical studies have concentrated on how PD patients perceive time compared to controls (a preliminary conclusion might be that temporal perception is variable, as even normal, daily experience corroborates: why can the same interval of veridical time seem to pass so quickly or so very slowly?).

More importantly, why does time perception matter in the first place? Mixed results in clinical research do not quite address the “why” question, which has been more fully examined in animal work. One should not conclude that the variability in clinical data forces one to dismiss the idea of a temporal-perceptual disturbance in PD. DA-mediated time perception is most obvious in scenarios where motor planning and associated reward are studied in a Pavlov-inspired experimental design. In human study, an important therapeutic implication is that current therapy to spare DA, or to enhance DA agonism, or the long-term, high-frequency stimulation of non-SNc sites may not rectify the inherently dynamic capacity of DANs in the pre-disease state. Loss of dopaminergic temporal dynamism has wide ranging network effects. DA denervation in PD represents both and output and input problem: not only is there loss of DA innervation of relevant pathways to striatum, thence to downstream sites, but also the role of DANs in afferent integration is compromised—the latter may be associated with time misperceptions associated with the disordered planning and execution of movement, not only in parkinsonism, but perhaps also in other movement disorders not associated specifically with DAN loss in SNc.

## Author Contributions

EM, with much advice from others noted in the acknowledgment section, conceived and conducted the review, wrote, and revised the article.

## Conflict of Interest

The author declares that the research was conducted in the absence of any commercial or financial relationships that could be construed as a potential conflict of interest.

## Publisher's Note

All claims expressed in this article are solely those of the authors and do not necessarily represent those of their affiliated organizations, or those of the publisher, the editors and the reviewers. Any product that may be evaluated in this article, or claim that may be made by its manufacturer, is not guaranteed or endorsed by the publisher.
