# Supplementary material for: Review: Subjective Time Perception, Dopamine Signaling, and Parkinsonian Slowness
Source: Front Neurol. 2022 Jul 8;13:927160. doi: 10.3389/fneur.2022.927160 (PMC9311331; doi:10.3389/fneur.2022.927160)
Supplement: Supplementary file 1 [file Table_1.docx]

Supplementary material.

Table 1. Explicit Timing Studies, listed in chronological order of publication (all vs. controls, with other intergroup analyses, as indicated)

| **Ref.** | **Method(s)** | **Clinical context(s)** | **Duration(s) examined** | **Summary Observations** |
| --- | --- | --- | --- | --- |
| 33 | time estimation (subject presented an interval, asked to estimate duration) and several tests of time reproduction (indicate when a prespecified period has passed) | OFF meds, then ON | 3-27 sec for time estimation tasks | overestimation of short time intervals (for both estimation and reproduction) when OFF |
| 28 | synchronization of finger tapping to target frequencies delivered by sound | ON meds | 1-3 hertz (Hz) vs. 5 Hz | PD patients too rapid at 1-3 Hz; too slow at 5 Hz |
| 29 | synchronization of finger tapping to target frequencies delivered by sound; continuation of tapping without sound | ON meds | sounds presented every 550 msecs | slight, but nonsignificant difference in those with a parkinsonism |
| 53 | time estimation; time production | OFF meds | 10, 30, 60 secs | PD patients underestimate intervals in estimation task and overestimate in production tasks |
| 16 | self-paced, repetitive finger tapping | ON meds vs. OFF; worse side vs. better side in asymmetrical PD | intertap target duration of 550 msecs | ON and OFF meds, inter-tap interval significantly shorter, especially ON  meds vs. controls; in asymmetric disease, tendency to tap too quickly on *both* sides |
| 30 | paced finger tapping to sound, then a duration perception task (determining the longer of two tones) | ON meds | separation of tones by 300  msecs and 600 msecs | variability in tapping intervals greater and duration perception error when interval between tone pairs was 600 msecs in PD patients |
| 17 | see text for discussion; peak interval timing procedure | OFF meds, then ON | 8, 21 secs | PD patients OFF meds late at 8 secs, early at 21 secs |
| 46 | variation on Malapani et al. (17) in which subjects were studied ON during training, then OFF during testing (then OFF training, ON testing) | ON and OFF meds | 6 and 17 secs | OFF training on testing both associated with migration (late at 6 secs, short at 17 secs |
| 43 | reproduction of a time interval presented visually | ON DBS of STN,  other  combinations of  ON and OFF DBS  and meds | 5 secs, 15 secs | OFF DBS/OFF meds association with overestimation of 5 secs, underestimation of 15 sec;  improvement with DBS and with meds |
| 49 | reproduction of time intervals presented visually, then production of the same intervals | ON meds | 5, 14, 38 secs | underestimation of longer durations; greater variability for 5-sec reproduction |
| 36 | theoretical paper, but included because of analysis of non-scalar properties in parkinsonian time estimation | ON and OFF  meds for training; then ON and OFF for testing | 6 and 17 sec | OFF for training and OFF for testing associated with non-scalar property (greater variability at 6 secs) |
| 55 | visual and auditory stimuli; subjects asked whether intervals were closer to 100 or 500 msec or 1 or 5 secs | ON meds | 100-500 msecs;  1-5 secs | significant impairment for auditory and visual temporal judgments in the 1-5-sec range |
| 34 | time production, reproduction | ON and OFF meds | 30-120 msecs (production);  250-2,000 msecs (reproduction) | nonscalar variance both ON and OFF meds in reproduction task |
| 54 | time reproduction (short and long intervals tested in inter-mingled and non-mingled fashion) | ON and OFF meds | 500-2,000 msecs | no difference for shorter intervals  (ON and OFF) compared to controls; underestimation of longer intervals when PD patients process different durations in a single task |
| 37 | time production, categorization of intervals (short vs. long) | ON and OFF meds | < 1 sec | two parkinsonian subgroups: one with high (nonscalar) variability across all tasks, another approximating controls |
| 51 | time production | OFF meds, compared to presymptomatic  and symptomatic Huntington’s disease | 1.2 secs | no differences between old normals, symptomatic and presymptomatic Huntington’s disease, and PD patients OFF meds |
| 41 | Study of metronome-based repetitive movements (finger tapping and drawing a circle, either one or continuous drawing of circles) | ON and OFF meds | 550 msec intervals | Temporal variability (coefficient of variation) did not differ between ON and OFF meds; no difference from controls |
| 45 | reproduction of duration of (self-paced) action | ON DBS of  STN/OFF meds; OFF DBS/OFF  meds; OFF DBS/ON meds | <1 min | under-reproduction of duration of action OFF DBS/OFF meds (but no impairment in ability to reproduce time intervals per se); improvement with either DBS or meds |
| 67 | auditory, visual stimuli/time reproduction; functional imaging study | ON and OFF meds | 1.2 or 1.8 sec stimulus duration; 24 secs total for a trial | lower accuracy OFF meds, visual worse than auditory; striatal dysfunction during encoding and decision periods compared to controls |
| 44 | time production, reproduction | OFF meds; frequency dependent modulation by DBS of STN | 5, 15 secs | PD: underestimation at 15 sec; reduced underestimation at 130 Hz DBS of STN, but not at 10 Hz |
| 38 | review, but included for comment about time production errors in suprasecond range | ON and OFF meds | 30-120 secs | significant impairment with loss of scalar property |
| 50 | time production, reproduction | review of PD and other conditions | 5, 38 secs | corroboration of migration effect–short interval judged longer, long interval shorter (time mis-estimations in epilepsy, brain injury as well) |
| 74 | duration-based timing, beat- based timing, deviation from isochrony in a 5-tone task (detection of disrupted metrical pattern) | STN of DBS  ON, then OFF, then ON again | intervals varied between tests, e.g., duration based timing 300-600 msecs | no differences for any task according to DBS status |
| 67 | unimodal and crossmodal signal processing (e.g., visual- visual; visual-auditory stimuli, comparisons of duration, functional imaging study | OFF meds | delay between standard & comparison intervals was 1.2-1.5 secs | PD: progressive underestimation of intervals in crossmodal comparisons; enhanced cerebellar connectivity in crossmodal tasks |
| 61 | time production | ON and OFF meds; multiple system atrophy (MSA)  comparison group | <5 secs, 5-15  secs, >15 secs. | MSA: underestimation of intervals >15 secs (no effect of levodopa); PD: overestimation of shorter intervals, <5 secs, correction with levodopa |
| 57 | time production and reproduction correlated with striatal DA transporter (DaT) deficits | ON meds | target durations 0.5-  300 secs | time underestimation for intervals >10 sec (in production, not reproduction tasks); striatal DaT deficit correlated with production underestimation |
| 71 | temporal bisection | likely ON meds, but not specified | 400 msecs,  1600 msecs | corroboration of migration effect–short interval judged longer, long interval shorter especially when visual stimuli expressed anger or shame |
| 69 | temporal bisection and trisection | ON meds | bisection (330  and 750 msecs); trisection (200,  550, 900  msecs) | impulsive decision strategies, bias to premature responses, especially in the more demanding trisection task |
| 70 | temporal bisection and foreperiod tasks | ON meds | in bisection task, 400 and  1600 msec standards; foreperiod varied from 1-3 secs | PD: normal foreperiod and sequential effects; shorter perceived durations and higher variability in temporal bisection tasks |
| 75 | time production (with stopwatch training for one month) | ON meds | 10 sec interval to be produced | after training, improvement in time production, in Go-No Go tasks, Stroop testing, and in impulsivity assessment |
| 66 | time production in those with cognitive dysfunction, electroencephalographic (EEG) correlation | ON meds; a subset studied ON and OFF meds | 3- and 7-sec intervals | PD: attenuated midfrontal ~4 Hz EEG activity at cue onset (start of the time production task) |
| 72 | temporal bisection, time production, reproduction | ON meds, but progressive supranuclear palsy (PSP) OFF levodopa also studied | anchor durations 400  and 1,600 msecs; production & reproduction 2-  8 sec intervals | subjective sense of 1 sec longer in PD, underestimation of long, suprasecond durations; see text for findings in PSP |

Table 2. Implicit Timing Studies, listed in chronological order of publication (all vs. controls, with other intergroup analyses, as indicated)

| **Ref.** | **Method(s)** | **Clinical context(s)** | **Duration(s) examined** | **Summary Observations** |
| --- | --- | --- | --- | --- |
| 76 | latency of response to delayed visual stimuli (three tasks), vocal answers | 1-3 weeks post- thalamotomy | delay of either 10 or 60 secs | 10 or 60 sec delays did not have effect in parkinsonism |
| 77 | Visual evoked potential (VEP) study in which different frequencies of visual stimuli are presented | PD treated and untreated | Normal VEP latency <116 msec (normal in early PD), but additional analysis performed based on potentials evoked by competing stimuli | Abnormality in VEP interactions at different frequencies |
| 31 | warning signal before a task, different motor response tasks | ~10 hours OFF meds, then ON | warning 0-3,200 msecs before task | no advantage with warnings after 200 msec either ON or OFF |
| 78 | words presented visually; subjects asked whether words had been seen previously and which word had been seen most recently | ON meds;  comparison group of subjects with Alzheimer’s disease | words presented every 2 secs | “recency deficit” in parkinsonism; content recognition deficit at short intervals between stimulus and test (0-2 intervening presentations of words) |
| 32 | a comparison of PD patients and other populations, including those with cerebellar disease | ON and OFF meds | not specified; discussion of hypothetical clock to time 400 msecs | only cerebellar patients showed a deficit in both the production and perception of timing tasks |
| 15 | temporal discrimination threshold for paired sensory stimuli | ON and OFF meds | minimal/initial time separation: 8 msecs  (auditory), 5 msecs (visual and tactile) | impaired temporal discrimination associated with parkinsonian clinical severity; ameliorated partially ON meds |
| 79 | temporal discrimination task: subject asked to determine the longer of two “empty” auditory intervals | ON meds | differences judged in relationship to a 50-msec standard interval | controls able to discriminate auditory intervals differing by 20 msecs;  PD patients discriminate only when intervals are at least 90 msecs apart |
| 80 | temporal discrimination task similar to Rammsayer and Classen (79) | a pharmacologic study of non- parkinsonian, normal subjects | 50-msec standard interval, compared in relation to 1,000- msec interval | haloperidol associated with impaired discrimination of 50-msec and 1,000- msec intervals (midazolam impaired discrimination only at 1 sec) |
| 40 | temporal estimation (visual) over 1 to several secs; then verbal estimation of duration of long intervals (12, 24, 48 secs) | ON meds | in the short- interval task, either 200 msec or 1 sec stimuli vs. four longer stimuli | PD patients less accurate in the short term; normal time estimation in longer term |
| 82 | temporal order discrimination; see text for discussion | ON meds | stimulus duration varied from 100- 700 msecs | PD patients underperformed at all durations <600 msecs |
| 83 | inspection time (IT); see text for discussion | ON and OFF meds | mean IT 169 msecs vs. 105  msecs (controls) | meds produced no significant effects in PD patients |
| 84 | foreperiod before cued action; see text for discussion | OFF meds | 1, 2.5, 4, 6.5 sec  foreperiods | no effect of foreperiod on voluntary reaction |
| 68 | timing cues in control of gait | ON and OFF meds | 60, 80, 100  steps/min | PD patients either ON or OFF with “locked” step length regardless of external cues |
| 108 | temporal discrimination; see text for discussion | OFF meds | <200 msecs | discrimination impaired OFF meds |
| 109 | temporal discrimination | in multiple system atrophy, not PD | 5-msec intervals between stimuli to determine threshold | correlation between severity of parkinsonism and abnormal increases in temporal discrimination threshold |
| 110 | temporal discrimination (threshold and  temporal order) of visual, tactile, and visuo- tactile stimuli | PINK1 heterozygotes without parkinsonism compared to symptomatic subjects | interval between stimuli in 10-  msec steps from 0-400 msecs | higher temporal discrimination thresholds of tactile and visuo-tactile stimuli in unaffected heterozygous carriers |
| 64 | review, but included because of its discussion of contingent negative variation (CNV), a slow, negative potential shift observed in averaged EEG data | normals (see Pfeuty et al., 2005) | < 2 secs | decreased amplitude of CNV in PD, restoration ON meds |
| 59 | seven test conditions, including three threshold discriminations | ON and OFF meds | 1,100 msec gap between stimuli in threshold studies | no marked deficit parkinsonian stimulus timing either ON or OFF meds compared to controls |
| 65 | review, but included because of discussion of event related potential (ERP, viz., P600, at 600 msec) in auditory processing of sentences | ON meds | <600 msec (before P600 ERP) | reduced P600 in PD patients ON meds; see also 62 |
| 60 | Predictions regarding a moving target in PD and degenerative cerebellar disease | ON meds for PD group | 1 sec presentation time | Impaired velocity estimation of a moving target in PD; prediction of moving target’s terminus in space impaired in cerebellar disease |
| 62 | Event related potential (ERP) study, correct vs. incorrect sentences presented aurally | ON meds | P600 peak at ~ 0.6-1.0 sec | Impaired late (P600) processing, preserved early processing in PD |
| 58 | Auditory processing study, ascertainment of duration of gaps in continuous noise, but a second test in which in which continuity of noise varied between trials, and study of what happens at the moment the continuity changes | OFF meds and DBS of STN, then ON meds, then ON STN of DBS | Variable gap duration, starting at 50 msec | Deficit in detection of gaps in PD, with incomplete rectification with STN of DBS (no rectification with meds); but, in second test, no difference between PD and controls |
| 111 | Somatosensory temporal discrimination | OFF/ON meds and OFF/ON meds in subgroup of gait freezing | Threshold higher in all PD (mean 127 vs. 78 msec) | Severity of gait freezing correlated with higher thresholds; improving in threshold on meds for all PD |
| 112 | somatosensory temporal discrimination | ON DBS of  STN/ON meds; ON meds/OFF DBS; meds OFF/DBS ON;  meds; OFF DBS/OFF meds | thresholds in PD (OFF  meds/OFF DBS) greater than controls by 60-80  msecs | medications, but not DBS of STN, improves somatosensory discrimination thresholds studied in three areas of the body |
| 93 | synchronization and continuation motor tasks to auditory cues, positron emission tomography (PET) study | ON  (apomorphine) and OFF meds | 50 msecs per tone, interstimulus interval of 1 sec | OFF meds: cerebellar activation more prominent, less frontal activation |
| 94 | visuospatial memory task | OFF meds | 500 msec memory array | PD: impaired filtering of distracting aspects of an array image |
| 95 | predictive motor timing tasks | OFF and ON meds, but 50% drug naive | button push every 2.5 to 4.5 secs | PD: “trouble postponing” action until the proper moment |
| 113 | temporal discrimination threshold; PET study | ON and OFF meds, PET OFF meds | prolongation of threshold ~39 msec | PET uptake tracks with prolonged threshold, but not with all PD rating scale subscores |
| 90 | threshold for detecting change in velocity of a moving image | ~4 hours after last dose | threshold for perceived velocity change  ~75 msecs in PD and controls | unchanged velocity perceived as acceleration (acceleration bias) less in PD, related to degree of bradykinesia |
| 96 | acoustic startle stimulus vs. “go” stimulus | ON and OFF meds | “premotor” reaction time (RT) ~173  msecs | shortening of premotor RT by startle whether ON or OFF meds |
| 97 | synchronization to tone sequences; positron emission correlation | ON meds, then placebo pill | tones separated by 500, 1,000,  1,500 msecs | *better* synchronization accuracy with  *greater* striatal DA denervation |
| 98 | visual smooth pursuit tracking task in forward direction then in reverse | early PD, most still drug naive | target moves for either 1,200  or 2,400 msecs | anticipatory eye movements less frequent in PD, but timing of anticipation matched controls |
| 99 | synchronization of movement to a beat | ON meds | intervals between sounds 1.5 or  2.5 secs | poor synchronization related to severity of PD, not to spatial constraints of the task |
| 105 | study of movement perception; perceived temporal durations of a visual presentation in two parts–e.g., upright vs. inverted | OFF meds | presentation durations of a second part, ranging from - 900 msecs to  +900 msecs,  compared to 1- sec first presentation | “temporal dilation” (e.g., tendency to perceive an upright motion as lasting longer) reduced in PD |
| 114 | somatosensory temporal discrimination, then real and sham continuous theta burst stimulation of cerebellum (cTBS) | OFF meds | interstimulus intervals in 10- msec steps | cTBS improved temporal discrimination only in PD off meds (only on the side of cerebellar burst stimulation) |
| 100 | temporal order judgment (TOJ, subjects asked which of two images appears first) | ON meds | variable interval depending on correct responses, beginning at 117 msecs | TOJ impaired in PD and in healthy elders |
| 101 | beat or rhythm discrimination | ON/OFF not specified | 4 tests used, different beat intervals | PD: association between impaired perception of rhythm and tests of verbal working memory and focused attention |
| 115 | temporal discrimination threshold (TDT); inertial study of speed, amplitude, and frequency of finger tapping | OFF meds; 24  of 33 patients were drug naive (non-tremor subjects) | TDT longer in PD by ~50 secs | Longer TDT associated with reduced mean values and higher variability in amplitude, speed, and frequency of finger tapping |
| 89 | visual identification of two targets with distractor stimuli (“attentional blink” AB: tendency not to observe second target when presented soon after first target | ON and OFF meds | stimuli presented at 10 Hz  frequency | OFF meds, overall poor target identification (both); wide variability among OFF patients (some never identify second target); correction with levodopa but not dopamine agonist |
| 91 | threshold study while listening to original, then altered music | ON meds | delay time interval 80-300 msecs | no difference in “just noticeable difference,” but impaired detection of 220-300 msec intervals |
| 92 | somatosensory temporal discrimination with kinematic analysis | ON and OFF meds | interstimulus intervals in 10- msec steps | PD: abnormalities in the temporal coupling between tactile information and motor outflow |
| 102 | perception of rhythmic regularity vs. remembered temporal associations | ON meds, comparison group with cerebellar ataxia | target intervals of 600 and 900 msecs | PD: impairment in rhythm-based temporal predictions  Cerebellar disease: impairment in interval-based temporal predictions. |
| 25 | somatosensory temporal discrimination | OFF meds; essential tremor and dystonia also studied | interstimulus intervals in 10- msec steps | discrimination thresholds higher in PD and dystonia, normal in essential tremor |
| 108 | visual saccade task in PD and PARKIN PD | ON and OFF meds (for PD subjects) | target stimulus 50 msecs each; intertarget foreperiod 400-  1900 msecs | influence of antecedent foreperiod significant in controls and PARKIN patients, but not in PD (perseverative tendency), either ON or OFF meds |
| 103 | vibrotactile stimulation, inhibition of effect of transcranial magnetic stimulation (TMS); functional imaging study | ON and OFF meds | long latency afferent inhibition (LAI), duration of sensory stimulation 200-1000  msecs | reduced LAI ON and OFF meds; deficient activation of contralateral primary sensory cortex and reduction in sensorimotor integration (less effect of TMS over primary motor cortex, suggesting deficient somatosensory processing) |
| 73 | threshold study (minimum needed to differentiate from a standard stimulus) | ON and OFF meds, ON and OFF DBS of STN | standard visual stimuli 0.5 and  1 sec, various comparison durations | no difference from control for the shorter (0.5 sec) stimulus in OFF state; higher threshold for 1 sec stimulus in PD OFF either meds or DBS of STN and PD ON either meds or DBS of STN |
| 107 | subjective estimation of time used to examine works of art | ON meds | “as long as they wanted” | ALL (PD and controls) overestimated time used, less overestimation in PD; shorter objective examination times in PD, regardless of complexity of art |
| 87 | variable foreperiod task with “imperative” stimulus during foreperiod | ON and OFF meds | short foreperiod mean 1.5 secs;  long 3 secs | imperative stimulus introduced temporal uncertainty; PD OFF associated with impaired use of prior information which corrected to control values when ON meds |
| 85, 86 | subjective rating of rhythm complexity at varying tempos | ON meds | 90, 120, 150  beats/minute (bpm) | PD: higher complexity ratings at 120 and 150 bpm, not at 90 bpm |
| 104 | perceived timing of a past event intended to trigger action | ON and OFF meds | random delay to trigger, 1-2 secs | PD: sensory event triggering an action perceived to have occurred earlier in time |
| 66 | time production in those with cognitive dysfunction, electroencephalographic (EEG) correlation | ON meds; a subset studied ON and OFF meds | 3- and 7-sec intervals | PD: attenuated midfrontal ~4 Hz EEG activity at cue onset (start of the time production task) |
| 88 | use of temporal orienting cues in different discrimination tasks | OFF meds in a first experiment then ON and OFF meds in a second | foreperiods after initial cue: 540 or 1,580  msecs | first experiment: OFF meds, impaired use of cues in the context of distractions; second experiment: ON and OFF meds, subjects benefitted from cues, similar to controls |
